# Prevalence and socio-behavioral influence of early childhood caries, ECC, and feeding habits among 6 – 36 months old children in Uganda and Tanzania

**DOI:** 10.1186/1472-6831-12-24

**Published:** 2012-07-26

**Authors:** Ray Masumo, Asgeir Bardsen, Kijakazi Mashoto, Anne Nordrehaug Astrom

**Affiliations:** 1 Department of Clinical Dentistry, Community Dentistry, University of Bergen, Bergen, Norway; 2 Centre for International Health, University of Bergen, Bergen, Norway; 3 Muhimbili University of Health and Allied Sciences, Dar Es Salaam, Tanzania

## Abstract

**Background:**

Early childhood caries (ECC) is a serious problem that has remained unexplored in sub-Saharan Africa**.** This study aimed to identify possible socio-behavioral correlates of ECC focusing 6–36 months old children and their caretakers.

**Methods:**

Cross sectional studies were conducted in a high fluoride rural area, Manyara, Tanzania and a low fluoride urban area, Kampala, Uganda. Totals of 1221 and 816 child - caretaker pairs attending health care facilities for growth monitoring were recruited in Manyara and Kampala, respectively. All caretakers completed face to face interviews at the health care facility. Children underwent oral clinical examination whereby ECC and Enamel hypoplasia were recorded using the dmft (WHO 1997) and the DDE index (FDI 1992).

**Results:**

The prevalence of ECC was 3.7% in Manyara and 17.6% in Kampala. According to multiple logistic regression analyses, received oral health information from health worker was the strongest determinant of ECC in Manyara, adjusted OR 0.3, 95% CI 0.09 – 0.93. In Kampala, visible plaque, high sugar intake and presence of enamel hypoplasia associated with ECC, adjusted ORs 2.8 (95% CI 1.61- 4.95), 3.0 (95% CI 1.39 – 6.34) and 2.3 (95% CI 1.36 - 3.95).

**Conclusion:**

Oral health education aimed at caretakers of 6–36 months, including health care workers’ information regarding the detrimental consequences for oral health of frequent sugar consumption and poor oral hygiene is important for prevention of ECC in Tanzania and Uganda.

## Background

Early childhood caries (ECC) is a serious oral health problem, especially in socially disadvantaged populations [1-4]. According to the American Academy of Paediatric Dentistry, ECC denotes any form of caries (cavitated or non- cavitated) occurring in the primary dentition of children aged 71 months or younger [5]. In the literature, various terminologies of ECC have been used, such as baby-bottle tooth decay, nursing caries and nursing bottle syndrome, indicating that feeding habits play a significant role as risk indicator in caries development of infants and youngsters [5,6]. The terminology of ECC reflects its multifactorial characteristics, whereby microbiological, socio-demographic, psycho-social and behavioral risk factors contribute to the development of caries lesions [6]. In spite of being largely preventable, ECC has remained relatively unexplored in many developing countries including sub-Saharan Africa [2-6]. Left untreated, ECC impacts on the quality of life to an extent similar to other systemic diseases and might lead to dental pain, avoidance of certain types of foods and might interfere adversely with anthropometric and nutritional status, socializing, self-esteem and learning abilities [7,8]. Initiation of preventive programs before or soon after tooth eruption seems necessary to optimize the chances of infants and youngsters to stay free of dental caries [9].

Whereas studies conducted in developed countries report a substantial reduction in the prevalence of dental caries in the permanent dentition of school aged children, there are no conclusive evidence regarding the trends in the primary dentition [10]. A comprehensive review including studies from Europe, Africa, Asia, the Middle East and North America revealed that the prevalence of ECC in socially disadvantaged groups globally could be as high as 70% [5]. In England and the United States of America the prevalence of ECC has been documented to vary between 7.0% and 12.0% and 11% and 53.1%, respectively [1]. Zhou et al. [3] reported a prevalence of ECC of 26.6% among 2 year old children in Southern China. Kumarihamy et al. [4] reported a prevalence of ECC in 1–2 year- olds in Sri Lanka of 32.1%. In 2004, Kiwanuka et al. [11] found a prevalence of ECC amounting to 45% in 3- year- old Ugandan preschool children. Kerosuo and Honkala [12] compared the caries experience of Finnish and Tanzanian children in 1991 and found that the proportion of maxillary incisors affected by caries was nearly three times higher in Tanzanian compared to the Finnish children. In 1994, Matee et al. [13] reported the prevalence of ECC among 1–4 years old to range between 1.5% and 12.8% in 9 regions of mainland Tanzania.

A recently published multi-level conceptual model, incorporating influences of ECC exerted at the individual, family and community level suggests that both social- and behavioral change is important in the prevention of this oral disease [14]. Some studies have shown that the lower the social class, in terms of low parental education, poor household income and lack of dental awareness, the worse children’s oral health status [14]. Other studies have disconfirmed such relationships [15,16]. Diets containing highly fermentable carbohydrates (sweets, ice cream and soda) have been associated with increments of ECC in a number of studies [17,18]. Others have not confirmed this relationship [11,13]. Evidence suggests that teeth are susceptible to ECC shortly after tooth eruption and prior to final maturation. This is the time when the child is breastfed. Feeding habits such as breastfeeding above one year and breastfeeding at night beyond tooth eruption may be associated with ECC [19]. According to a systematic review, however, evidence does not suggest a consistent and strong association between breastfeeding and ECC, thus research examining this relationship has been equivocal [20]. Moreover, evidence suggests that the presence of visible plaque and developmental enamel defects increases the risk of the initiation and progression of ECC [6,21,22].

In sub-Saharan Africa, globalization has been linked with change from a traditional- to a western-style diet. Between 1991 and 2005 per capita sugar consumption (kilogram’s per year) in Tanzania and Uganda increased reportedly from 4.4 to 7.2 and from 2.4 to 9.7 [23]. Sugar consumption among pre-school children has been associated with mother’s level of education and household income [11,17]. Previous studies document mothers as role models for their pre-school children in establishing food preferences, including intake of sugared snacks and drinks [24,25]. World Health Organization (WHO) has called against unhealthy dietary messages to children, since advertising and marketing of sugary foods and beverages contribute to sugar consumption in developing countries [26]. Unhealthy radio and/or television messages have influenced mothers or caregivers to feed their children with sweets, to add sugar to complementary foods and drinks, to use sweets as rewards, to allow sweet consumption during the day and to spend more money on sweets [4,26,27].

Detailed information regarding the prevalence and influencing factors of ECC provides a valuable tool in the planning, implementation and evaluation of oral health promotion programs. Such evidence is rare when it comes to infants in Tanzania and Uganda. Focusing 6–36 month olds and their caretakers from high- and low fluoridated areas of Tanzania and Uganda, this study aimed to assess the frequency and socio-behavioral distribution of infants’ ECC and sugar consumption.

## Method

### Manyara site

Recruitment of children and their primary caretakers took place at Haydom Lutheran Hospital (HLH) and in its 20 mobile outreach community service sites in Mbulu, Hanang and Babati districts of Manyara region, northern Tanzania, from August 2010 to January 2011. Manyara region covers an area of 50,836 km^2^ and has a population of 1,037,605 of whom 18% are children below 5 years of age. The region is populated with a predominately poor rural population with a literacy rate of 73% [28]. It is administratively divided into 5 districts, three of which, Mbulu (4452 km_,_^2^ total population 237,280), Hanang (3899 km_,_^2^ total population 204,640) and Babati (7169 km^2^, total population 302,253), constituted the study areas [29]. The districts have drinking water with fluoride content about 3.0 mg F/L [30]. In collaboration with the government authorities through District Health Management Teams (DHMT) and other Voluntary Agencies, HLH has taken responsibility for an extensive outreach programme covering Reproductive and Child Health (RCH) services [31]. The RCH includes one post with daily activities at the hospital in addition to 20 community posts, visited once a month by car on a rotating basis. The community outreach posts are not health facilities but may be in any building available in the respective villages. According to the 2002 population and housing census in Tanzania, the RCH outreach programme covered 6 out of 54 villages in Hanang, 3 out of 81 villages in Babati and 12 out of 70 villages in Mbulu, serving respectively, 4790, 1538 and 7910 children below 5 years of age [29]. During the project period, 21 RCH outreach posts were visited 3–5 times on a rotating basis, recruiting 10–14 caretaker-child pairs per visit. All caregiver-child pairs who were resident in the catchment areas of the RCH posts and who satisfied the inclusion criteria of being a mother or primary caregiver of children aged 6–36 months attending for immunization and/or growth monitoring, were invited to participate in the study. Mothers were the primary target respondents, but in case of mothers’ absence, the primary caregiver was recruited. Out of 1250 caregiver-child pairs approached, 1221 agreed to participate (total response rate 97.7%, range 94-99%). A sample size (n = 1221) of this magnitude is sufficient to the pre-calculated sample size of 810 caregiver-child pairs, assuming a prevalence of ECC of 50%, a margin error of 5%, confidence level of 95% and an assumed design effect of 2. Another 5% was added to the sample size to account for non- response and children that were excluded because they were the second eligible child of the same mother or caregiver.

### Kampala site

A cross-sectional Reproductive and Child Health (RCH)-based study was conducted in Kampala district, Uganda, from June to October, 2007. Kampala covers an area of 197 km^2^ and has a population of 1.2 million of whom 18% are under 5 years. Kampala has an overall literacy rate of 75% [28]. It is administratively divided into 5 divisions, two of which, Nakawa (42.5 km^2,^ total population in 2008 300,000) and Makindye (40.6 km^2^, total population in 2008 380,000), constituted the study areas. The districts have drinking water with fluoride content about 0.3 mgF/L [32]. One non-governmental (Kibuli) and one governmental (Naguru) RCH care facility were purposely selected in Makindye and Nakawa, respectively. Both facilities have large catchment areas and include community outreach clinics for the provision of child immunization. The inclusion criteria were caregivers with children aged 6–36 months attending the Kibuli and Naguru clinics for immunization and/or growth monitoring. All caregiver-child pairs who attended the clinics during the study period and who satisfied the inclusion criteria predefined for the study were eligible for participation. Out of 831 caregivers approached, 816 agreed to participate (response rate 98%). This satisfied a pre-calculated sample size assuming a prevalence of ECC of 30%, a standard error of 3% and a confidence interval of 95%. Another 5% was added to the sample size to account for children who had to be excluded from analysis for being the second eligible child of the same mother/caregiver. For a more detailed description of the sampling procedure, see [33].

### Ethical clearance

In Tanzania, permission was granted by Medical Research Coordinating Committee of Ministry of Health and Social Welfare and the Ethical Research Committee in Norway (REK VEST). In Uganda, permission was given by The Ethical Committee of Uganda National Council of Science and Technology, Research and Publication Committee at Makerere University. Informed written consent was obtained from participating caregivers in both recruitment sites. When the caregivers could not read and write, verbal consents were obtained.

### Clinical oral examination

Clinical examinations were carried out by trained and calibrated dentists (RM in Manyara and JK in Kampala), whereas trained assistants recorded the observations. Calibration exercises were carried out according to the guidelines on training and calibration published by British Association of the study of Community Dentistry (BASCD) [34]. Children were examined in knee to knee position using a dental mirror and natural light. The total number of teeth present was initially recorded and dummy variables for upper and lower jaws were constructed (0) = 0–4 teeth and (1) = 5–10 teeth. Visible plaque was recorded as (0) absent and (1) present. Teeth were cleaned and dried by sterile gauze and inspected for ECC and enamel hypoplasia using disposable dental mirrors. Dental caries was assessed on fully and partially erupted teeth according to the World Health Organization criteria and recorded in terms of decayed, filled and missed teeth [35]. No radiographs were taken and decay was recorded at the level of cavitation. In the present analysis, decayed teeth (dt) was used as dependent variable, both dichotomized as (0) absent (dt = 0) and (1) present (dt =1) and used as a count variable. Enamel hypoplasia was recorded on the buccal surfaces of each tooth present according to the criteria described by the developmental defects of Enamel (DDE) index proposed by FDI [36]. Experience with enamel hypoplasia was recoded as (0) absent (DDE = 0) and (1) present (DDE > 0).

### Interview

An interview schedule was constructed in English and translated into Luganda and Kiswahili, the main languages in Kampala and Manyara. Kiswahili is the national official language in Tanzania spoken proficiently by almost 95% of Tanzanians. Single words of the Kiswahili interview was translated into Iraque, Datoga, Nyaturu and Nyisanzu languages, when deemed necessary during the interview. The interview schedule was translated in several steps; from English into local languages by bi-lingual Kiswahili/English and Luganda/English professionals, and then back translated to English by independent translators. Project staff at both sites reviewed the interview schedule for semantic, experiential and conceptual equivalence to the original version. Sensitivity to culture and selection of appropriate words were considered. The interview schedule was piloted among caregivers of primary school children to evaluate the quality of the translations in terms of comprehensibility, readability and relevance to assess face validity. At both sites, a slightly modified interview schedule was administered in the field by trained locally recruited research assistants. The interviews were performed in face to face interviews with primary caretakers before their children underwent oral clinical examination. *Sugar consumption* was used as both dependent and independent variable in the analyses and was assessed by asking “have you ever given child (Name) glucose water, sugar water, milk tea with sugar, black tea with sugar, sweet potatoes, sugared soda, biscuits/cakes/ice-cream, and sweets/toffees/chocolate in the past 24 hours? Responses were given as (0) No and (1) Yes. A sum score was constructed (range 0–8, median 4.0) and dichotomised using the median split into 0 = low intake and 1 = high intake. *Teething symptoms* was assessed by asking “Did (Name) experience the following symptoms (gum irritation, fever, loss of appetite, diarrhea, increased salivation, vomiting, convulsions, coughing) during his/her tooth eruption? Responses was given as (0) No and (1) Yes. A sum score was created (range 0–8) and dichotomized yielding 0 = less symptoms (score ≤3) and 1 = more symptoms (score >3). *Dental awareness* was assessed by asking, “Have you ever received information from health worker/radio on how to care for your own teeth and gums?” Responses were given as (0) No and (1) Yes. *Current breastfeeding* were assessed in terms of yes (1) and no (0). *A measure of breastfeeding duration* was achieved by asking mothers who confirmed breastfeeding “For how long have you breastfed*”. Socio-demographic variables* were assessed in terms of age and sex. Mother’s and fathers education were assessed by asking; “What is the highest level of school you/father of the child have attended? Responses were given as (0) No formal education, (1) Did not complete primary school, (2) Completed primary school, (3) Secondary, (4) Completed Secondary, (5) College/University. A dummy variable was constructed 0 = lower education and 1 = at least primary education. *Family wealth* was assessed as an indicator of socio-economic status according to a standard approach in equity analysis [37]. Durable household assets indicative of family wealth (i.e. radio, television, telephone, refrigerator, lantern, cupboard, bicycle, motor cycle, car, boat) were recorded as (0) “not available and/or not in working condition” or (1) “available and in working condition.” These assets were analyzed using principal components analysis (PCA). The first component resulting from this analysis was used to categorize households into four approximate quartiles of wealth ranging from the 1^st^ quartile (least poor) to the 4^th^ quartile (poorest).

### Statistical analyses

Predictive Analytics SoftWare (PASW) version 18.0 was used for data analysis. Design effect was adjusted using generalized linear models for binominal distributions in STATA (version 10.0). Univariate analyses were performed by use of chi-square statistics. Multiple variable analyses were performed using logistic regression with odds ratios (OR) and 95% confidence intervals (CI) and Poisson regression with rate ratios (RR). Since using dummy variables runs the risk of loosing information, results from logistic regression analyses were checked using Poisson regression with count variables.

## Results

### Reliability

In Manyara, the agreement between the examiner and the gold standard on the DDE- score was Cohen’s kappa 0.82. Duplicate examinations were performed with 80 child-caregiver pairs 3 weeks apart. In Kampala, duplicate examinations involved 20 child-caregiver pairs 3 weeks apart. Test-retest reliability of caregiver’s report on child’s sugar intake, in terms of Intra class correlation coefficients (ICC) was 0.96 (95% CI 0.94 – 0.98) in Manyara. Corresponding figure in Kampala was 0.80 (95% CI 0.59 – 0.91). In Manyara, intra-examiner reliability in terms of Cohen’s kappa for dental caries and enamel hypoplasia ranged from 0.85 to 1.0 and from 0.91 to 0.97, respectively. In Kampala, Cohen’s kappa for both dental caries and enamel hypoplasia ranged from 0.8 -1.0. Thus, acceptable levels of intra-examiner agreement (kappa > 0.60) was obtained at both study sites [38].

### Sample characteristics and descriptive analyses

A total of 816 (94.6% mothers, mean age 24.7 years, SD 4.7) and 1221 (98.6% mothers, mean age 28.3 yrs, SD 6.5) caregiver/child pairs participated in Kampala and Manyara, respectively. Table 1 depicts the frequency distribution of participants by socio-demographic-, behavioral- and clinical variables by study site. Seventy eight percent and 71.8% of participating mothers reported at least primary education in Kampala and Manyara, respectively. One half and one third of the responding mothers in Kampala and Manyara were below 24 years of age. Corresponding rates for current breastfeeding were 57% and 60%. At both study sites, about half of the participating mothers confirmed children’s mouth cleaning on a regular daily basis, whereas about one quarter and one half had received oral health information from health workers and radio, respectively. About half of the caregivers in Manyara and Kampala confirmed children’s sugar consumption to be above the median on the sugar sum frequency score. The mean dt score and significant caries index (SiC index) in Kampala was 0.73 and 2.20. The corresponding figures in Manyara were 0.08 and 0.24, respectively (not in table). As shown in Table 1, the prevalence of ECC (dt > 0) amounted to 17.6% in Kampala and 3.7% in Manyara.

**Table 1:** Frequency distribution of study participants according socio-demographic, behavioral and clinical variables across Kampala (n = 816) and Manyara (n = 1221)

**Variables**	**Categories**	**Kampala % (n)**	**Manyara % (n)**
Sex of child	Boy	50.7 (414)	50.5 (616)
	Girl	49.3 (402)	49.5 (605)
Age of child	6-12 month	45.5 (371)	29.6 (362)
	13-24 months	29.7 (242)	50.9 (621)
	25-36 months	24.9 (203)	19.5 (238)
Household assets index	1^st^ quartile-least poor	24.0 (185)	26.8 (327)
	2^nd^ quartile	24.2 (187)	25.0 (305)
	3^rd^ quartile	26.0 (210)	24.8 (303)
	4^th^ quartile- poorest	25.8 (199)	23.4 (286)
Mother’s education	Lower	22 (176)	28.2 (344)
	At least primary	78 (624)	71.8 (877)
Father’s education	Lower	7 (49)	25.6 (309)
	At least primary	93 (654)	74.4 (899)
Caretaker’s age	<24 yrs	54.3 (443)	33.8 (403)
	25 yrs and above	45.7 (373)	66.2 (789)
Teeth present upper	0-4	56.3 (435)	55.6 (679)
	5-10	43.7 (337)	44.4 (542)
Teeth present lower	0-4	56.3 (434)	55.4 (677)
	5-10	43.7 (337)	44.6 (544)
Plaque score	No	62.6 (483)	40.1 (490)
	Yes	37.4 (289)	59.9 (731)
Cleaning mouth	More seldom	50.4 (389)	53.8 (657)
	At least daily	49.6 (383)	46.2 (564)
Breast feeding	No	42.7 (330)	39.8 (486)
	Yes	57.3 (442)	60.2 (735)
Sum score of dietary sugar intake	Low	36.3 (280)	54.9 (670)
	High	63.7 (492)	45.1 (551)
Received inform from Health Worker	No	77.5 (632)	80.6 (984)
	Yes	22.5 (184)	19.4 (237)
Received inform from Radio	No	40.0 (309)	33.0 (403)
	Yes	60.0 (463)	67.0 (818)
Enamel hypoplasia	No	85.9 (663)	92.1 (1124)
	Yes	14.1 (109)	7.9 (97)
Teething symptoms	Less	48.5 (304)	47.7 (539)
	More	51.5 (323)	52.3 (590)
Caries experience	dt = 0	82.4 (636)	96.3 (1176)
	dt > 0	17.6 (136)	3.7 (45)

### ECC distribution by tooth type

**Figure 1:**
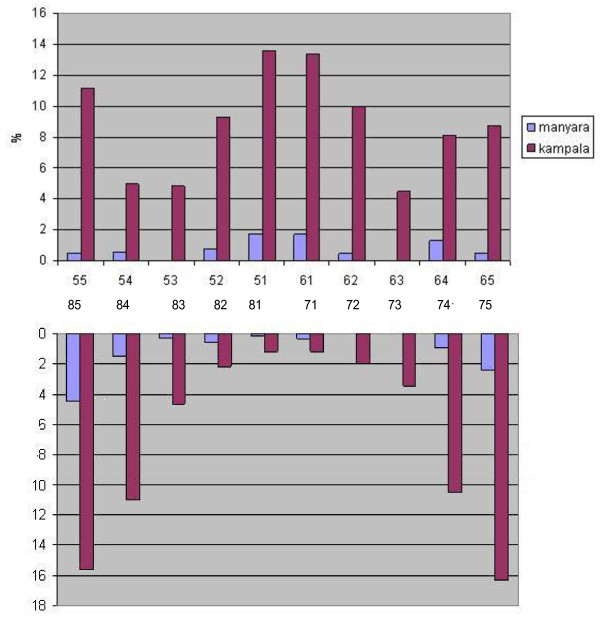
Frequency distribution of caries experience according to tooth type in Manyara, Tanzania (high fluoridated rural area) and Kampala, Uganda (low fluoridated urban area).

Table 2 depicts caries experience across various groups of teeth (i.e. anterior maxillary, anterior mandibular and molars) by age, sex and breastfeeding status. An increase in the ECC by increasing age was observed for all teeth groups, except for anterior mandibular teeth in Manyara. Across study sites and teeth groups, ECC varied inversely with current breastfeeding status. Totals of 1.2% of the breastfed children versus 7.4% of the weaned children (p < 0.001) presented with ECC in Manyara. Corresponding figures in Kampala were 6.3% and 33% (p < 0.001). Nevertheless, the mean number of months having breastfed was reportedly 19.6 (sd 5.7) in children with caries and 16.9 (sd 5.6) (p < 0.001) in children without caries experience in Manyara. In Kampala, the corresponding figures were 18.1 (sd 6.5) and 13.0 (sd 6.9) (not in Table). As shown in Figure 1, the tooth-specific pattern of caries was similar across study sites. Maxillary central incisors and mandibular molar teeth were the most frequently affected teeth both in Manyara and Kampala. However, the caries rates were highest among children in Kampala for each tooth type. The frequency distribution of caries experience according to tooth type and sex in Kampala showed that girls had consistently higher caries experience than boys across all teeth. A less consistent pattern was observed in Manyara where boys showed higher caries experience than girls in 52, 62, 84 and 74.

**Table 2:** Any caries experience (dt > 0), and caries experience in anterior maxillary, anterior mandibular and molar teeth (upper and lower) by age group and breastfeeding status sex in Manyara and Kampala

	**Any caries % (n)**	**Anterior maxillary % (n)**	**Anterior mandibular % (n)**	**Molar caries % (n)**
***Manyara***				
6 -12 months	0.8 (3)	0.0 (0)	0.6 (2)	0.3 (1)
13- 24 months	2.3 (14)	1.1 (79	1.0 (69)	0.8 (5)
25 – 36 months	11.8 (28)**	5.0 (12)**	0.4 (1)	7.1 (17)**
Breastfeeding; yes	1.2 (9)	0.4 (3)	0.8 (6)	0.4 (3)
Breastfeeding: no	7.4 (36)**	3.3 (16)**	0.6 (3)	4.1 (20)**
***Kampala***				
6 -12 months	2.2 (6)	2.2 (6)	0.0 (0)	0.0 (0)
13- 24 months	12.9 (29)	12.1 (27)	0.0 (0)	3.1 (7)
25 – 36 months	53.2 (101)**	36.8 (70)**	12.1 (23)**	34.2 (65)**
Breastfeeding: yes	6.3 (28)	4.8 (21)	0.2 (1)	2.5 (11)
Breastfeeding: no	33.0 (109)**	24.5 (81)**	6.7 (22)**	18.5 (61)**

* P < 0.05, **P < 0.01.

### Socio-demographic and behavioral factors associated with ECC and sugar intake

All socio-demographic-, behavioral-, and clinical variables that were statistically significantly associated with ECC and the sum score of sugar consumption in univariate analyses (P < 0.05) were included in multiple variable logistic regression analyses and Poisson regression analyses (Table 3 and Table 4). According to initial univariate analyses, sex of child, household assets, parental education and symptom status were not statistically significantly associated with ECC, whereas children’s sex and caretakers’ age did not associate significantly with sugar consumption. The final logistic regression model for Manyara presented in Table 3 revealed that having 5–10 teeth present in upper jaw and having a high sugar consumption associated with higher odds of having ECC, [OR 8.9 (95% CI 1.52-52.47) -1.8 (95% CI 1.00-3.49)]. Having received oral health information from health care worker was associated with lower odds of having ECC [OR 0.3 (95% CI 0.09-0.93)]. In Kampala, older children, having visible plaque on anterior teeth, having enamel hypoplasia and having a higher sugar consumption associated with higher odds of ECC [OR 5.2 (95% CI 1.31-21.01) -2.3 (95% CI 1.36-3.95)]. The following variables, that were statistically significantly associated with ECC in the univariate analyses, did not maintain their significant relationship in the multiple variable model; age of child, plaque score, status of breastfeeding, enamel hypoplasia in Manyara and teeth present in upper jaw, teeth present in lower jaw, plaque scores, status of breast feeding in Kampala.

**Table 3:** Distribution of ECC by socio-demographic and behavioral factors, Manyara (n = 1221) and Kampala (n = 816)

***Manyara***	**Categories**	**% (n)**	**Adjusted OR (95% CI)**	**Adjusted RR (95% CI)**
Age of child	6-12 month	0.8 (3)	1	0.2 (0.05 – 0.78)
	13-24 months	2.4 (14)	0.4 (0.06 - 2.95)	0.3 (0.19 – 0.55)
	25-36 months	11.8 (28)**	1.2 (0.15 – 8.92)	1
Teeth present upper jaw	0-4	0.4 (2)	1	0.2 (0.06 – 0.42)
	5-10	5.6 (43)**	8.9 (1.52 -52.47)	1
Plaque score	No	1.0 (5)	1	1.9 (0.98 – 3.49)
	Yes	5.5 (40) **	1.2 (0.35 – 3.77)	1
Current breastfeeding	No	7.4 (36)	1	1.0 (0.51 – 1.79)
	Yes	1.2 (9) **	0.8 (0.30 – 2.17)	1
Enamel hypoplasia	No	3.2 (36)	1	0.7 (0.41 – 1.16)
	Yes	9.3 (9)**	1.6 (0.72 – 3.61)	1
Sum score of dietary sugar intake	Low	2.2 (15)	1	0.6 (0.37 – 0.88)
	High	5.4 (30) **	1.8 (1.00 – 3.49)	1
Received information from health care worker	No	4.3 (42)	1	2.2 (1.11 – 4.38)
	Yes	1.3 (3)*	0.3 (0.09 – 0.93)	1
***Kampala***				
Age of child	6-12 month	1.9 (7)	1	0.3 (0.09 – 1.22)
	13-24 months	12.8 (31)	1.4 (0.39 - 4.69)	0.5 (0.29 – 0.78)
	25-36 months	54.2 (110)**	5.2 (1.31 – 21.01)	1
Teeth present upper jaw	0-4	1.1 (4)	1	0.4 (0.09 – 1.68)
	5-10	31.4 (144)**	2.7 (0.62 – 12.09)	1
Teeth present lower jaw	0-4	1.9 (7)	1	0.5 (0.11 – 2.13)
	5-10	32.1 (141)**	2.2 (0.47 – 10.36)	1
Caretakers age	> 24 years	14.7 (65)	1	0.8 (0.60 – 1.19)
	25 yrs and above	22.3 (83)**	1.3 (0.83 – 2.12)	1
Plaque score	No	5.2 (25)	1	0.5 (0.29 – 0.78)
	Yes	38.4 (111) **	2.8 (1.61 – 4.95)	1
Current breastfeeding	No	33.0 (109)	1	0.9 (0.53 – 1.49)
	Yes	6.3 (28) **	1.4 (0.70 – 2.79)	1
Enamel hypoplasia	No	13.3 (88)	1	0.7 (0.48 – 0.98)
	Yes	44.0 (48)**	2.3 (1.36 – 3.95)	1
Sum score of dietary sugar intake	Low	3.6 (10)	1	0.5 (0.24 – 0.91)
	High	25.6 (126)**	3.0 (1.39 – 6.34)	1

* P < 0.05, **P < 0.01.

**Table 4:** Children’s sugar intake by socio-demographic and behavioral factors

***Manyara***	**Categories**	**% (n)**	**Adjusted OR (95% CI)**	**Adjusted RR (95% CI)**
Age of child	6-12 month	29.8 (108)	1	0.8 (0.72 - 0.92)
	13-24 months	49.1 (305)	0.5 (0.29 - 0.86)	0.9 (0.92 - 1.09)
	25-36 months	58.0 (138) **	1.1 (0.71 – 1.59)	1
Mother’s education	Lower	35.8 (123)	1	0.9 (0.89 - 1.02)
	At least primary	48.8 (428) **	1.2 (0.90 - 1.65)	1
Father’s education	Lower	34.6 (107)	1	0.9 (0.87 – 1.01)
	At least primary	48.6 (437)**	1.3 (0.95 - 1.76)	1
Household assets index	1^st^ quartile-least poor	32.5 (93)	1	0.7 (0.72 - 0.85)
	2^nd^ quartile	37.0 (112)	1.1 (0.79 – 1.67)	0.8 (0.75 – 0.89)
	3^rd^ quartile	50.5 (154)	1.9 (1.33 – 2.81)	0.9 (0.83 – 0.97)
	4^th^ quartile- poorest	58.7 (192) **	3.1 (2.11 – 4.51)	1
Teeth present upper jaw	0-4	38.3 (260)	1	1.0 (0.93 – 1.09)
	5-10	53.7 (291)**	1.0 (0.67 - 1.42)	1
Cleaning mouth	More seldom	37.4 (246)	1	0.9 (0.85 – 0.97)
	At least daily	54.1 (305) **	1.7 (1.29 – 2.25)	1
Breast feeding	No	55.8 (271)	1	1.1 (0.98 – 1.16)
	Yes	38.1 (280)**	0.8 (0.54 - 1.12)	1
Received information from Radio	No	35.5 (143)	1	0.9 (0.85 – 0.97)
	Yes	49.9 (408)**	1.3 (1.02 – 1.79)	1
Teething symptoms	Low	39.9 (215)	1	0.8 (0.84 – 0.95)
	High	50.7 (299)**	1.7 (1.31 - 2.19)	1
***Kampala***				
Age of child	6-12 month	43.0 (154)	1	0.8 (0.75 – 1.00)
	13-24 months	73.2 (164)	1.6 (0.98 – 2.80)	0.9 (0.88 – 1.07)
	25-36 months	91.6 (174)**	4.3 (1.67 – 11.01)	1
Mother’s education	Lower	68.6 (216)	1	1.0 (0.93 – 1.06)
	At least primary	60.6 (275)*	0.7 (0.50 – 1.12)	1
Teeth present upper jaw	0-4	46.9 (204)	1	0.9 (0.82 – 1.02)
	5-10	85.5 (288)**	1.7 (0.93 – 3.20)	1
Cleaning mouth	More seldom	49.4 (192)	1	0.9 (0.86 – 0.98)
	At least daily	78.3 (300)**	2.6 (1.76 – 3.95)	1
Breast feeding	No	82.4 (272)	1	1.0 (0.93 – 1.11)
	Yes	49.8 (220)**	0.7 (0.40 – 1.09)	1
Received information from Health worker	No	66.6 (401)	1	1.1 (1.04 – 1.23)
	Yes	53.5 (91) **	0.4 (0.28 – 0.73)	1
Teething symptoms	Low	64.5 (196)	1	0.9 (0.89 – 1.02)
	High	74.3 (240)**	1.6 (1.09 – 2.37)	1

* P < 0.05, **P < 0.01.

As shown in Table 4, the final logistic regression model revealed that in Manyara, belonging to the poorest household quartile, performing mouth cleaning at least daily, having received health information from radio and having high score on symptom index were associated with higher odds of having high sugar consumption [OR 3.1 (95% CI 2.11-4.51) - OR 1.3 (95% CI 1.02-1.79)]. In Kampala, older age groups and mouth cleaning at least daily were associated with higher odds of sugar consumption [OR 4.3 (95% CI 1.67-11.01) - OR 2.6 (95% CI 1.76-3.95)], whereas having received information about oral health from health care workers was associated with lower odds for having high sugar scores (OR 0.4 (95% CI 0.28-0.73). The following variables that were statistically significantly associated with sugar consumption in the univariate analyses, did not influence sugar consumption in the multiple variable analyses; age of child, mothers’ education, fathers’ education, teeth present in upper jaw, breastfeeding status in Manyara and mothers’ education, teeth present in upper jaw, status of breast feeding in Kampala. Breastfeeding status discriminated between children being high and low on the sum sugar consumption score. Whereas 56% of children not breastfed and 38% of children breastfed presented with higher sugar consumption in Manyara (p < 0.001), the corresponding figures in Kampala were 82% and 45% (p < 0.001). Poisson regression confirmed the results from multiple variable logistic regression analysis presented in Table 3 and 4.

## Discussion

This is one of the first population based studies to systematically investigate correlates of ECC and sugar consumption among infants 6–36 months old residing in rural high fluoridated- and urban low fluoridated regions of Tanzania and Uganda. Thus, the present study provides information about age groups that have not been covered by the national oral health surveys in Tanzania and Uganda. Information about the prevalence of ECC in the general pediatric population of sub-Saharan Africa is scarce and the Kampala and Manyara region have been surveyed to a very limited extent [5,6]. Many previous studies regarding determinants of ECC and sugar consumption have focused solely on individual factors studied by univariate analyses in relatively small samples [11-13]. In contrast, this study included various potential risk indicators using multivariable models, young and well defined age groups and large samples from different regions of sub-Saharan Africa. A comparison of the Manyara study group with the pediatric population (0–4 yr) in Mbulu, Babati and Hanang districts on socio-demographic markers, revealed that the study participants was representative of 6–36 month olds resident in those areas. In Kampala, the sampling method might make the external validity more questionable. However, high response rates and a limited number of missing items in the interview suggest that the study group for whom there are complete data reflects caregiver/child pairs living in the catchment areas of the RCH clinics in Makindye and Nakawa districts.

The prevalence of ECC in Manyara (3.7%) and Kampala (17.6%), reflected neglect of infants’ oral health, suggesting that caries might be a public health problem in the two areas [8]. Although evidence of a major benefit of fluoride consumption in infancy is limited, the discrepancy in caries experience between the study sites indicated that living in the high fluoride area of Manyara might be recognized as a caries protective factor [39]. Consistent with previously reported geographical variations in ECC, the present one is most probably attributable to various fluoride levels in drinking water [40]. Nevertheless, it is difficult to evaluate the global amount of fluoride (systemic and topical) to which children are exposed. Widespread use of fluoride toothpaste has been associated with a significant reduction of caries [6], however, the affordability and availability of fluoride toothpaste in its bioactive form is still a main challenge in many areas of sub-Saharan Africa [41]. Care should be exercised when directly comparing findings between Manyara and Kampala, considering possible confounding due to different time points of study conduct. Confounding is also possible due to differences between examiners for whom the inter-examiner consistency was not known, although both examiners were trained and calibrated according to the same rules at University of Bergen, Norway. In both study sites, decayed teeth were the only component contributing to the dmft scores, reflecting unmet treatment needs due to poor accessibility of oral health care services in both countries. The present findings accord with rates of ECC assessed in studies of similar age groups in Southern China, Sri Lanka and Nigeria [3,4,42] and also with studies conducted in the Western world [39]. Moreover, the prevalence of ECC documented here is consistent with that reported decades ago in Tanzanian children aged 1–4 years [13], but slightly lower than the rate reported among 3–5 yr olds in South Africa and Uganda [2,11]. The various diagnostic and epidemiological criteria employed across studies make it difficult to obtain a clear picture of ECC rate across time and separate regions of sub-Saharan Africa. A possibility of misclassification between ECC and dental fluorosis in Manyara should also be considered.

**Figure 2:**
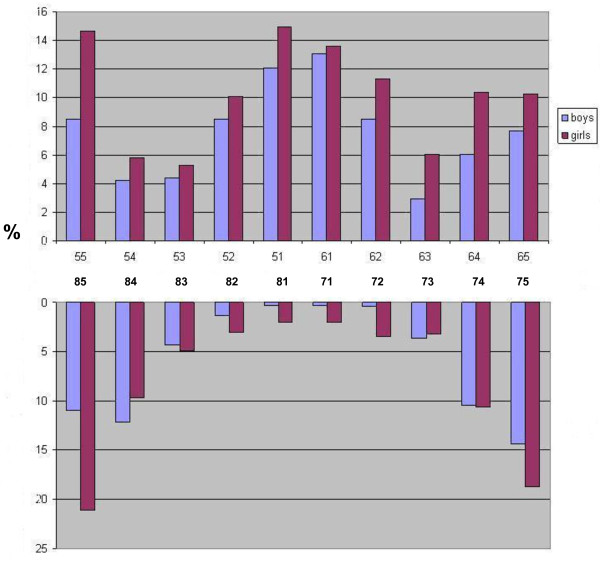
Frequency distribution of caries experience according to Sex and tooth type in Kampala, Uganda (low fluoridated urban area).

**Figure 3:**
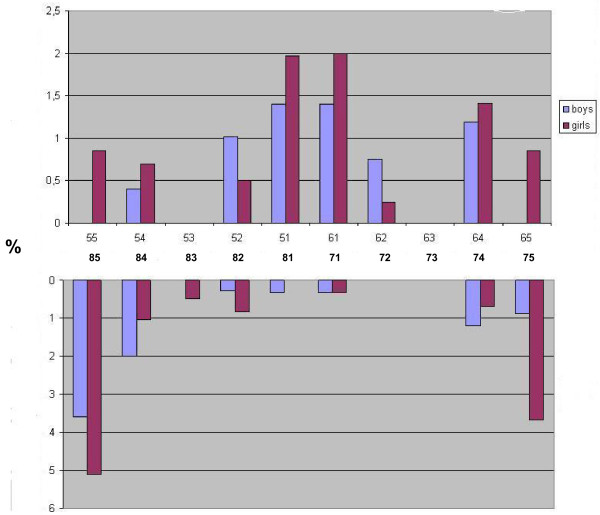
Frequency distribution of caries experience according to Sex and tooth type in Manyara, Tanzania (high fluoridated rural area).

As depicted in Figures 1, 2, 3, the caries lesions were not evenly distributed, being most prevalent in the upper maxillary anterior teeth and the lower molars for both boys and girls, thus reflecting the patterns of eruption. Similar caries patterns have been reported in previous surveys of the primary dentition [11,12] and have been attributed to infant feeding practices; baby bottle filled with sweetened beverages and prolonged breastfeeding on demand [43]. This study revealed an on average longer breastfeeding exposure among children with than without caries experience. Previous evidence suggests that prolonged breastfeeding beyond one year is associated with ECC [20,44,45]. On the other hand, the prevalence of ECC recorded in the present study varied inversely with current breastfeeding status, being highest among children who were not breastfed across tooth types and study sites (Table 2). Moreover, weaned children presented with higher sugar consumption than their breastfed counterparts, indicating a protective effect of breastfeeding on ECC, as reported previously [46]. Nevertheless, breastfeeding status did not maintain its significant relationship with ECC in multiple variable analyses (Table 3). Being associated with both sugar consumption and ECC, the effect of breastfeeding was probably hidden in the final regression model, mediated or confounded by sugar consumption or other variables.

Consistent with a number of studies, but at odds with others, the present study revealed a positive association between sugar consumption and ECC in Manyara and Kampala [17,47]. A previous study of 3-5- yr- old preschool children in Kampala did not identify a similar relationship [11]. In Kampala, the rates of both ECC and sugar consumption increased significantly with increasing age suggesting children’s adoption of adverse dietary habits and cariogenic foods when growing older. Previous evidence suggests that caregivers of 0–23 months-old Ugandan infants add sugar to complementary foods and drinks more often than using oils and milk [27]. Sugar intake of 6-36- month olds increased with increasing teething symptom status suggesting a higher caries risk in diseased children consuming medicinal syrups for a longer time as has been reported previously [48,49]. Advertisements and marketing of sweets and sugary beverages have influenced caregivers towards feeding their children with sweets and snacks [26]. In contrast, this study revealed that caregivers who received oral health information from health care workers were less likely to have children with high sugar consumption and ECC. A recent review has shown that children of less well informed and lower educated parents have higher sugar intake leading to higher levels of dental caries [14]. These findings reflect important oral health consequences of early oral health information from health care workers provided to caregivers of 6–36 month old children in Tanzania and Uganda.

Poor oral hygiene practices have been found to be strongly associated with the prevalence of ECC [6,50]. In Kampala but not in Manyara, children with visible plaque on anterior maxillary teeth, recognized as a proxy of poor tooth brushing frequency, were more likely to have ECC as compared to their plaque free counterparts. This study also documented enamel hypoplasia as a potential risk indicator of ECC among Kampala children [21,22]. Enamel hypoplasia provides suitable sites for the adhesion and colonization of cariogenic bacteria. Evidently, ECC on such altered surfaces develop more rapidly than on sound tooth surface [21]. A low prevalence of ECC in Manyara due to high fluoride in drinking water might explain the lack of statistical significant relationship with visible plaque and enamel hypoplasia. Moreover, there is a risk of misclassification between enamel hypoplasia and dental fluorosis.

Previous studies have shown that socio-demographic factors such as parental education and household income have a direct influence on ECC [2,6,47]. Traebert et al. [51] reported a positive relationship between low maternal schooling and severity of dental caries in Brazilian preschool children. No such relationship was identified in this study, although belonging to the poorest household quartile emerged as a risk indicator for higher sugar consumption in Manyara. It is well known that determination of social class is a challenge in developing countries where accepted criteria for social classification do not exist [37]. Moreover, it should be considered that socio-demographic variables in line with the other self- reported variables utilized in this study might have been subject to recall and response biases [52].

## Conclusions

In sum, this study showed that ECC is present shortly after tooth eruption among infants in both high and low fluoridated areas of Manyara and Kampala. Moreover, this study confirmed the importance of dietary habits and oral hygiene for the ECC development, thus extending findings from developed countries to 6–36 month old children in Tanzania and Uganda. According to the present study, lack of information from health workers, high sugary food and beverage intake, presence of visible plaque and presence of enamel hypoplasia were risk indicators for ECC. ECC and sugar consumption were less prevalent in breastfed children, whereas duration of breastfeeding was longer in children with and without ECC. Thus the role of breastfeeding in protection of ECC remains equivocal. Sugar consumption was determined by low household income, experiences of teething symptoms and health information received by radio and health care workers. Future studies should assess risk indicators using longitudinal analyses. The findings presented can be utilized in the planning of oral health promotion programs that should start during pregnancy and the first months after birth in order to optimize ECC prevention in infants and youngsters.

## Competing interests

The authors declare that they have no competing interests.

## Authors’ contributions

RM: principal investigator, designed the study, collected the data (Manyara site), performed the statistical analyses, and wrote the manuscript. AB: participated in the design of the study and provide valuable guidance in the data collection and has been actively involved statistical analyses and writing the manuscript. KM: provide valuable guidance in statistical analyses. JK collected data (Kampala site). AN: main supervisor, designed the study, guided the statistical analyses and writing the manuscript. All authors have read and approved the final manuscript.

## Authors’ information

RM: PhD candidate, University of Bergen. AB: Professor, Department of Clinical Dentistry, University of Bergen, Norway. KM: PhD, Muhimbili University of Health and Allied Sciences, Dar Es salaam, Tanzania. AN: DDS PhD, Professor, Department of Clinical Dentistry, Community Dentistry, and Centre for International Health, University of Bergen, Norway.

## Pre-publication history

The pre-publication history for this paper can be accessed here:

http://www.biomedcentral.com/1472-6831/12/24/prepub
